# Antibiotic-Only Treatment of Pediatric Retropharyngeal and Parapharyngeal Abscess: A Case Report

**DOI:** 10.7759/cureus.93147

**Published:** 2025-09-24

**Authors:** Tao Li, Chen Yang, Xiuqin Hao, Yi Lou, Guangsheng Wu

**Affiliations:** 1 Pediatrics, Hangzhou Normal University Affiliated Hospital, Hangzhou, CHN; 2 Pediatrics, Hangzhou Rehabilitation Hospital, Hangzhou, CHN

**Keywords:** antibiotic treatment, children, deep neck infection, early diagnosis, retropharyngeal and parapharyngeal abscesses

## Abstract

Deep neck infections (DNIs) in children are rare and atypical in early clinical manifestations. They are frequently misdiagnosed or overlooked and have a rapid onset and can progress to life-threatening complications. There are only a few reports on pediatric DNIs’ clinical manifestations, diagnostic clues, and non-surgical treatment in China. This report presents a case of deep neck infection in a 9-year-5-month-old child that was successfully managed with intravenous antibiotics alone, without the need for surgical intervention. The patient initially presented with fever, sore throat, and hoarseness. Physical examination revealed ipsilateral neck swelling, head tilt, and restricted neck movement. Laboratory tests showed markedly elevated inflammatory markers, and neck CT revealed abscesses in the retropharyngeal and parapharyngeal spaces. The purpose of this case report is to emphasize the importance of early diagnosis and timely treatment to enable successful non-surgical management.

## Introduction

Deep neck infections (DNIs) are acute suppurative infections involving the potential spaces beneath the cervical fascia. The most common types include retropharyngeal, parapharyngeal, peritonsillar, submandibular, and multi-space abscesses, with retropharyngeal and parapharyngeal abscesses being the most frequent [1]. DNIs occur more often in children, with nearly two-thirds of cases reported in those under six years of age. The predominant pathogens are *Streptococcus pyogenes* and *Staphylococcus aureus*. Early symptoms are often nonspecific and resemble those of upper respiratory tract infections (URTIs), such as fever and sore throat. As the infection progresses, patients may develop dysphagia, trismus, neck stiffness, and localized swelling or masses [2,3], which can complicate timely diagnosis. Anatomically, the parapharyngeal space is located lateral to the pharynx, extends from the skull base to the hyoid bone, and has an inverted pear shape. The retropharyngeal space lies posterior to the pharynx and serves as a key pathway for infection spread into the mediastinum. Such infections can extend intracranially, resulting in cerebral abscess or meningitis [4], or descend into the mediastinum, leading to necrotizing mediastinitis, a condition associated with a mortality rate of 11%-40% [5,6]. First-line therapy typically consists of antibiotics, particularly cephalosporins or β-lactam/β-lactamase inhibitor combinations with anaerobic coverage. Surgical drainage is warranted in patients with systemic toxicity, airway compromise, large abscesses, or lack of clinical improvement after 48 hours of antibiotic therapy. This report describes a case of a combined retropharyngeal-parapharyngeal abscess, underscoring the importance of early diagnosis and timely intervention in preventing life-threatening complications.

## Case presentation

A 9-year-5-month-old boy was admitted on April 30, 2024, with a two-day history of fever, sore throat, and right neck swelling with pain. Initial symptoms included hoarseness, mouth breathing, and snoring. He had received oral cephalosporin for two days without improvement. Neck pain and swelling worsened, with head tilt and restricted movement. There was no drooling or trismus. Appetite, bowel, and urinary habits were normal. On admission, temperature was 38.0°C, pulse 111 bpm, respiratory rate 25/min, blood pressure 120/69 mmHg, and oxygen saturation 100%. Height was 149 cm, and weight was 38 kg. Examination revealed a tender, fixed, poorly defined mass in the right mid-neck. Lungs were clear. Laboratory tests showed WBC 13.9×10⁹/L, neutrophils 74%, serum amyloid A (SAA) >220 mg/L, C-reactive protein (CRP) 63.14 mg/L, and erythrocyte sedimentation rate (ESR) 73 mm/h. Ultrasound revealed multiple enlarged cervical lymph nodes, the largest 3.2×1.0 cm on the right.

**Figure 1:**
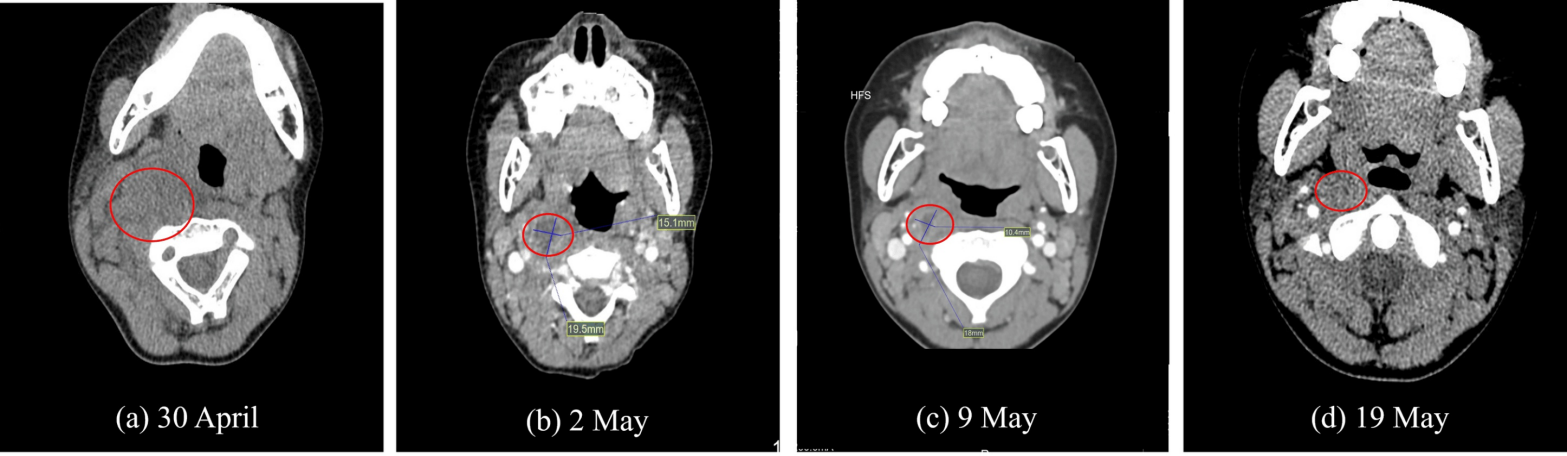
CT manifestations of parapharyngeal and retropharyngeal abscesses across different stages. (a) Neck CT plain scan showing the abscess at the site marked by the red circle. (b) Contrast-enhanced chest CT demonstrating a right retropharyngeal and parapharyngeal abscess measuring 19.5 × 15.1 mm (red circle). (c) Contrast-enhanced neck CT showing a right retropharyngeal and parapharyngeal abscess measuring 18.0 × 10.4 mm (red circle). (d) Contrast-enhanced cervical CT indicating significant absorption and reduction of the abscess at the site marked by the red circle, compared with the scan performed on May 9, 2024.

The initial diagnosis was acute tonsillitis with cervical lymphadenitis. The disproportionate swelling and limited mobility suggested deep neck infection. Contrast-enhanced CT showed multiple low-density retropharyngeal and parapharyngeal collections (Figure 1a). Thoracic CT confirmed a 19.5 × 15.1 mm abscess without mediastinal spread (Figure 1b). The final diagnosis was retropharyngeal and parapharyngeal abscess.

**Figure 2:**
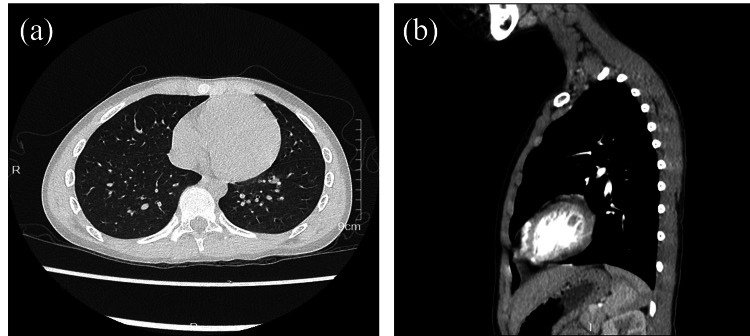
Contrast-enhanced chest CT findings showing no evidence of abscess formation. (a) Lung window: no abscess detected. (b) Mediastinal window: no evidence of mediastinal abscess formation.

The initial diagnosis was acute tonsillitis and acute cervical lymphadenitis. However, the disproportionate right neck swelling relative to lymph node size, restricted neck movement, and rapid clinical progression raised suspicion for a deep neck abscess. A neck CT scan revealed hypodense fluid collections with rim enhancement in the retropharyngeal and parapharyngeal spaces, consistent with abscess formation (Figure 1a). Following thoracic surgery consultation, contrast-enhanced chest CT confirmed an abscess (approximately 19.5 × 15.1 mm) within the right retropharyngeal and parapharyngeal spaces (Figure 1b), with no mediastinal involvement (Figure 2). The patient was definitively diagnosed with retropharyngeal and parapharyngeal space abscesses.

**Figure 3:**
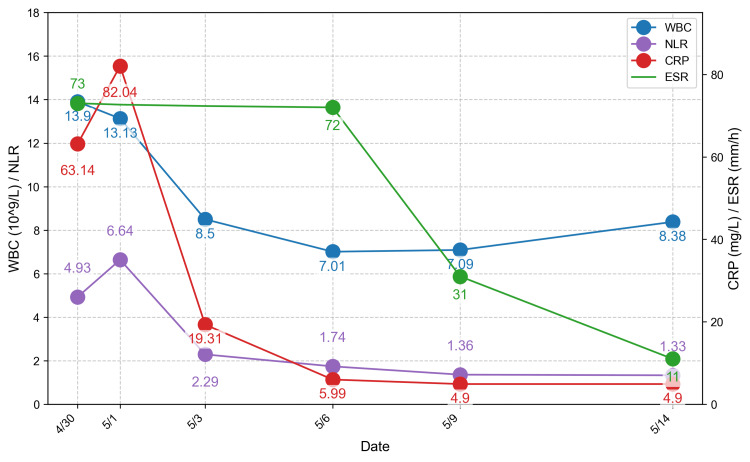
Trends in dynamic monitoring of inflammatory indicators in patients. WBC, white blood cell count; NLR, neutrophil-to-lymphocyte ratio; CRP, C-reactive protein; ESR, erythrocyte sedimentation rate.

**Figure 4:**
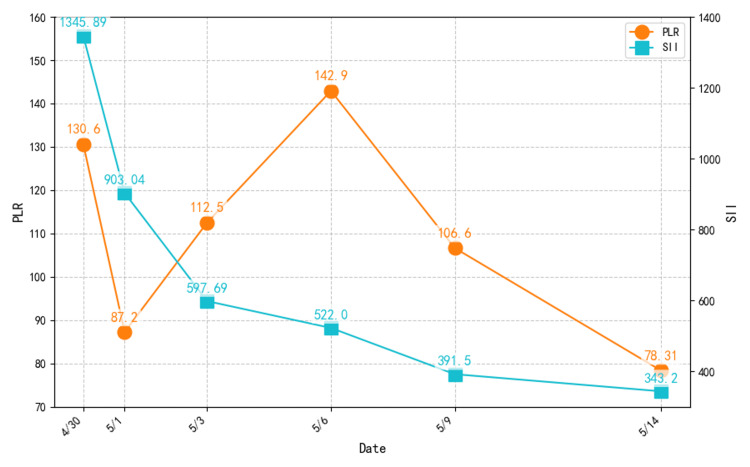
Dynamic changes of PLR and SII during hospitalization and treatment. PLR, platelet-to-lymphocyte ratio; SII, systemic immune-inflammation index.

Intravenous antibiotic therapy was initiated with amoxicillin-clavulanate at 90 mg/kg/day for 11 days (April 30 to May 11), along with a single dose of methylprednisolone sodium succinate at 1 mg/kg IV on May 1. Following diagnosis confirmation and otolaryngology consultation, meropenem (40 mg/kg/day IV) was added for eight days (May 1 to May 8) to broaden antimicrobial coverage. Serial otolaryngology evaluations, supported by contrast-enhanced neck CT and airway assessment, recommended continued medical management, with surgical drainage reserved for inadequate clinical response. Additional laboratory tests-including bacterial culture, EBV antibody and DNA testing, blood culture, and immune function-were unremarkable. However, an elevated anti-streptolysin O (ASO) titer of 1238 IU/mL indicated streptococcal infection. During treatment, pain gradually subsided, and serial inflammatory markers showed sustained decline (Figures 3, 4).

Body temperature normalized within three days. By day six, the neck mass, tenderness, and restricted mobility had resolved, accompanied by normalization of inflammatory parameters. Follow-up contrast-enhanced CT demonstrated a reduction in abscess size (Figure 1c), and the patient was discharged. A three-day course of oral amoxicillin-clavulanate was prescribed after discharge. Surveillance CT on May 19 showed near-complete resolution of the abscess (Figure 1d). During one year of follow-up, the patient remained clinically cured without recurrence.

## Discussion

DNIs are rare in pediatric patients but can progress rapidly. Delayed diagnosis and treatment may quickly result in severe complications. Therefore, children presenting with fever and a neck mass should be promptly and carefully evaluated. Early initiation of appropriate antibiotic therapy is critical for controlling infection and reducing the likelihood of surgical intervention. Drawing on clinical case studies, this study systematically summarizes and refines an effective diagnostic and treatment approach for pediatric DNIs. The proposed process emphasizes that diagnosis should be based on an integrated assessment of clinical symptoms, physical findings, inflammatory markers, and imaging studies. Management should prioritize antibiotic therapy, with adjunctive use of glucocorticoids when indicated, while surgical indications and timing must be assessed with caution. The following section provides a detailed discussion of this diagnostic framework and its clinical application.

Characteristic signs of suspected DNIs in children include neck swelling or mass, restricted or rigid neck movement, strabismus, neck pain, tooth clenching, anorexia, and deviation of the uvula [7,8]. Early clinical features often resemble those of common upper respiratory tract infections, typically beginning with fever, sore throat, or hoarseness. In infants and young children, nonspecific manifestations such as excessive salivation, refusal to feed, and irritability are common due to their limited ability to express symptoms [2]. In most cases, however, these characteristic signs appear only after the infection has breached anatomical barriers, and clinicians usually suspect DNIs only once such features are evident [2]. The prevalence and severity of symptoms vary widely among pediatric patients. Even when typical signs are present, cases may initially be misdiagnosed as lymphadenitis, tonsillitis, or infectious mononucleosis, leading to diagnostic delays or even missed diagnoses. Rare cases of parapharyngeal abscesses presenting with tongue protrusion have been reported [9]. Evidence suggests that the site of infection correlates with certain symptoms-for instance, neck pain is strongly associated with parapharyngeal and retropharyngeal infections [10]. Some children present only with fever at disease onset, and fever remains the most common clinical manifestation of DNI [11]. Therefore, DNI should be considered in the differential diagnosis of children with persistent fever, even in the absence of typical signs [1]. As the disease advances, more severe symptoms may develop, including respiratory distress, hoarseness, neck stiffness, and wheezing. Delayed diagnosis at this stage can result in life-threatening complications such as airway obstruction, mediastinitis, cavernous sinus thrombosis, or rupture of tracheal and pharyngeal abscesses [12].

When the clinical manifestations described above are present, diagnosis can be further supported by inflammatory markers. Among these, the neutrophil-to-lymphocyte ratio (NLR), CRP, platelet-to-lymphocyte ratio (PLR), and systemic immune-inflammation index (SII = NLR × platelets) are of particular importance due to their roles in risk stratification and prediction of complications. NLR is a sensitive marker of systemic inflammation. Baglam et al. reported that NLR≥5.4 predicts the risk of DNI secondary to acute bacterial tonsillitis [13]. Ban et al. found that NLR>8.02 significantly increased the likelihood of requiring surgical incision and drainage, particularly when accompanied by an ESR>56.6 mm/h [14]. Elevated NLR has also been associated with prolonged hospitalization, higher complication rates, and multiorgan involvement, with values >6.4 suggesting severe infection [14]. CRP rises rapidly in the early phase of infection. Levels >41.25 mg/L are associated with an increased risk of abscess formation, whereas values >156 mg/L indicate critical illness with a markedly elevated risk of complications [15]. PLR≥175 is linked to a higher risk of complications, with a negative predictive value of 98%. Similarly, SII >2975 is strongly predictive of severe outcomes, including mediastinitis, airway obstruction requiring tracheostomy, repeated surgical intervention, and even mortality [16]. Conventional markers also remain valuable: a neutrophil percentage (NEU%) >70-80% suggests active septic inflammation; procalcitonin (PCT) >0.5 ng/mL indicates bacterial infection, with values >2 ng/mL more suggestive of sepsis; and ESR >40 mm/h indicates prolonged disease or extensive infection (Table 1). Dynamic monitoring is essential: CRP and NLR typically decline significantly within 48-72 hours of effective treatment. Persistent elevation or further increases should raise concern for uncontrolled infection or disease progression.

**Table 1:** Inflammatory markers and their clinical significance. NLR, neutrophil-to-lymphocyte ratio; CRP, C-reactive protein; PLR, platelet-to-lymphocyte ratio; SII, systemic immune-inflammation index; NEU%: neutrophil percentage; PCT: procalcitonin; ESR, erythrocyte sedimentation rate.

Marker	Threshold/reference value	Clinical implication
NLR	≥5.4	Predicts risk of deep neck infection (DNI) secondary to acute bacterial tonsillitis
	>8.02 (with ESR >56.6 mm/h)	Higher likelihood of requiring surgical incision and drainage
	>6.4	Suggests severe infection with higher risk of complications
CRP	>41.25 mg/L	Increased risk of abscess formation
	>156 mg/L	Critical illness with markedly elevated risk of complications
PLR	≥175	Higher risk of complications; negative predictive value 98%
SII	>2975	Strongly predictive of severe outcomes, including mediastinitis, airway obstruction requiring tracheostomy, repeated surgical intervention, and mortality
NEU%	>70-80%	Indicates active septic inflammation
PCT	>0.5 ng/mL	Indicates bacterial infection
	>2 ng/mL	Strongly suggests sepsis
ESR	>40 mm/h	Indicates prolonged disease course or extensive infection
	>56.6 mm/h (with NLR >8.02)	Higher likelihood of surgical intervention

Definitive diagnosis of DNIs in children requires imaging studies. Contrast-enhanced computed tomography (CECT) is widely regarded as the gold standard [17]. With its high anatomical resolution and superior lesion delineation, CECT can accurately demonstrate the hypodense core and ring-enhancement typical of abscesses, differentiate cellulitis from abscess formation, and assess the extent of the infection. It is also invaluable for evaluating potential vascular complications, such as internal jugular vein thrombosis, aneurysm formation, or mediastinitis [18]. Despite its strengths, CECT has limitations. Although sensitivity is generally high, specificity is lower; for example, Daya et al. reported a sensitivity of 81% but a specificity of only 57%, with a notable proportion of cellulitis cases misclassified as abscesses [19]. To address this issue, Ban et al. proposed a clinical scoring system that integrates imaging findings (particularly rim enhancement) with inflammatory markers such as CRP, ESR, and NLR [14]. This combined approach achieved a sensitivity of 79.5% and a specificity of 82.8%, with rim enhancement identified as the most predictive CT feature of abscess formation. Alternative imaging modalities also have a role in selected cases. Ultrasound is well-suited for the initial evaluation of superficial infections but has limited utility in detecting deep-seated lesions. Magnetic Resonance Imaging (MRI) provides complementary information, particularly in the assessment of complications, but is rarely practical in emergency settings due to longer scan times, confined examination environment, noise, and the potential for poor cooperation from young children. These limitations contribute to a relatively high risk of examination failure in acute scenarios [20]. In cases of complex infections or those requiring multiple follow-ups, repeated imaging is essential. CECT offers high reproducibility, rapid acquisition, and consistent diagnostic performance. Particular attention must be paid to radiation exposure in pediatric patients. Techniques such as low-dose scanning, automatic exposure control, and iterative reconstruction can significantly reduce radiation dose while maintaining diagnostic accuracy [21]. In the emergency evaluation and follow-up of DNIs in children, low-dose CECT not only accurately depicts lesions and complications but also outperforms ultrasound and MRI in supporting timely and reliable clinical decision-making.

In the present case, the child initially exhibited symptoms consistent with an upper respiratory tract infection, including fever, sore throat, and hoarseness. Within a short time, however, neck swelling, tenderness, and restricted movement developed. Initial ultrasound suggested lymphadenopathy, leading to a provisional diagnosis of acute tonsillitis with cervical lymphadenitis. The possibility of DNI was not considered at that stage. On subsequent clinical examination, the swelling was found to be inconsistent with the distribution of lymph nodes visualized on ultrasound, prompting further investigation. On admission, inflammatory markers were elevated: WBC 13.9 × 10⁹/L, NLR 6.64, CRP 82.04 mg/L, and ESR 73 mm/h (Figure 3). They were all above reference thresholds, indicating acute bacterial infection and an increased risk of abscess formation. Clinical assessment supported this suspicion. Contrast-enhanced CT revealed ill-defined changes in the right retropharyngeal and parapharyngeal spaces, with a poorly demarcated mass-like lesion showing ring enhancement. The laryngeal ventricle and pyriform sinus were clear, both vocal cords appeared normal, and the cricoid and thyroid cartilages showed no evidence of destruction. A diagnosis of DNI was established, and high-risk complications such as mediastinal extension were excluded. An NLR >8.02 combined with an ESR >56.6 mm/h suggested a possible need for surgical intervention, prompting otolaryngology consultation for airway and complication assessment. Therefore, an otolaryngology consultation was obtained to evaluate airway status and possible complications. However, both PLR (87.2-142.9) and SII (1345.89 → 343.2) (Figure 4) remained below high-risk cutoffs, suggesting a low risk of complications. Based on this assessment, conservative treatment was initiated.

Once DNIs are diagnosed, management should be guided primarily by systemic antimicrobial therapy, while the decision to pursue surgical or percutaneous drainage depends on both the clinical response and imaging findings. For patients demonstrating satisfactory improvement with intravenous antibiotics and an abscess cavity measuring <22 mm, intensive anti-infective therapy with close monitoring is generally recommended. Conversely, when the abscess cavity exceeds 32 mm, conservative treatment proves ineffective, or imaging reveals high-risk features such as gas formation, rupture of the abscess cavity, or mediastinal extension, timely surgical or percutaneous drainage should be performed [11,17]. Antibiotic selection must be individualized, taking into account the common pathogens, the child’s age, abscess size, and disease severity. Continuous reassessment of the clinical and laboratory course is critical, with the aim of minimizing the need for surgical intervention and optimizing cure rates.

In the absence of drug susceptibility results, empirical antimicrobial therapy should be guided by epidemiological patterns and age-related differences in etiology. Retropharyngeal and parapharyngeal space infections predominate in children under six years of age, accounting for approximately two-thirds of pediatric cases [19]. In contrast, peritonsillar abscesses are most common in adults. The prominent development of cervical lymph nodes in infants and young children, combined with their susceptibility to infections, is considered the anatomical basis for the higher prevalence of retropharyngeal abscesses in this population [7]. The primary source of pediatric deep neck infections is upper respiratory tract infection (30.4%), which may extend to the deep neck spaces either by direct spread from otorhinolaryngologic foci or through lymphatic drainage. Odontogenic infections represent the second most common cause, accounting for 25.3% of cases [22]. Children <5 years: Retropharyngeal and parapharyngeal abscesses are predominant, frequently arising from pyogenic lymphadenitis secondary to upper respiratory tract infections. The leading pathogens are *Staphylococcus aureus* (including methicillin-resistant strains, MRSA) and *Streptococcus* spp. Adolescents and adults: Peritonsillar abscesses are most frequently observed, typically as complications of acute tonsillitis. *Streptococcus aureus* and anaerobic bacteria are the primary causative organisms. Children aged 6-10 years and older (with occasional exceptions in younger atypical cases): Other forms of deep neck infections are more common, often secondary to odontogenic infections or streptococcal pharyngitis. The predominant pathogens in this group are *Streptococcus* spp. [23] and anaerobic organisms, usually presenting as mixed aerobic-anaerobic infections [19].

The therapeutic approach adopted in this case is largely consistent with findings reported in the literature. Scholars in Taiwan have recommended amoxicillin-clavulanic acid or third-generation cephalosporins as first-line agents for pediatric DNI, in combination with metronidazole to provide anaerobic coverage, with subsequent adjustment based on culture and sensitivity testing [1]. Their study further reported that approximately 40.4% of children can be managed successfully with antibiotics alone; however, in children aged ≤15 months or with an abscess cavity >2.2 cm, the likelihood of treatment failure is significantly increased, and surgical intervention should be prioritized [1]. The decision to escalate to early surgery in this case would have been appropriate had such risk factors been present. Other studies have emphasized the use of β-lactam/β-lactamase inhibitor combinations (e.g., amoxicillin-clavulanate, ampicillin-sulbactam) or β-lactamase-resistant agents (e.g., cefoxitin, imipenem, meropenem) for severe or rapidly progressive cases [2]. This aligns with the present case, in which empiric therapy with amoxicillin-clavulanic acid was upgraded to meropenem during disease progression to expand antimicrobial coverage and address potential resistance. Adjunctive glucocorticoid therapy has also demonstrated clinical benefit. Several studies suggest that corticosteroids may shorten the course of illness and facilitate recovery [10,24]. Tansey et al. further reported that dexamethasone significantly reduced the need for surgical drainage [25]. In addition, retrospective data have shown that cefotaxime combined with rifampicin as a first-line regimen markedly lowered surgical intervention rates (7.5% vs. 32.1%, adjusted HR=0.21), with efficacy unaffected by age or abscess size. Nonetheless, when the abscess cavity exceeded 32 mm, the risk of pharmacologic treatment failure rose sharply (HR=8.5), necessitating surgical drainage [17]. These findings are highly consistent with the present case, in which timely surgical intervention was pursued due to the presence of a large abscess cavity.

The treatment plan in this case adhered to evidence-based recommendations. Initial empiric therapy with intravenous amoxicillin-clavulanic acid was promptly escalated to meropenem in response to disease progression and pathogen-related risk factors, thereby broadening antimicrobial coverage against resistant organisms. Glucocorticoids were added to mitigate local edema and reduce the risk of airway compromise. Through standardized anti-infective therapy, adjunct medication, and dynamic imaging monitoring, the patient’s clinical condition improved progressively, with gradual resolution of the abscess cavity (Figure 1). Furthermore, inflammatory markers showed significant improvement: WBC decreased to 8.38×10⁹/L, NLR sequentially declined to 1.8 and 4.9, and ESR dropped to 11 mm/h (Figure 3), ultimately leading to clinical cure. This treatment course not only demonstrates the rationality and effectiveness of the clinical strategy but also corroborates the practical applicability of relevant research findings.

## Conclusions

Based on previous literature, this study suggests that the early management of pediatric DNI should follow the principle of “early recognition-timely imaging evaluation-prompt antimicrobial therapy-close monitoring-graded intervention.” Treatment strategies should be guided by evidence-based medicine and individualized according to patient age, abscess size, and disease progression. Future prospective multicenter studies are warranted to validate the clinical benefits of different antibiotic regimens, define the appropriate population for corticosteroid use, and explore the role of imaging parameters in therapeutic stratification, with the aim of optimizing clinical pathways, improving outcomes, and reducing surgical intervention rates.

## References

[1] Huang CM, Huang FL, Chien YL, Chen PY (2017). Deep neck infections in children. J Microbiol Immunol Infect.

[2] Esposito S, De Guido C, Pappalardo M (2022). Retropharyngeal, parapharyngeal and peritonsillar abscesses. Children (Basel).

[3] Rigotti E, Bianchini S, Nicoletti L (2022). Antimicrobial prophylaxis in neonates and children undergoing dental, maxillo-facial or ear-nose-throat (ENT) surgery: a RAND/UCLA appropriateness method consensus study. Antibiotics (Basel).

[4] McDowell RH, Hyser MJ (2025). Neck abscess. StatPearls.

[5] De Palma A, Cantatore MG, Di Gennaro F (2022). Multidisciplinary approach in the treatment of descending necrotizing mediastinitis: twenty-year single-center experience. Antibiotics (Basel).

[6] Bali RK, Sharma P, Gaba S, Kaur A, Ghanghas P (2015). A review of complications of odontogenic infections. Natl J Maxillofac Surg.

[7] Chang L, Chi H, Chiu NC, Huang FY, Lee KS (2010). Deep neck infections in different age groups of children. J Microbiol Immunol Infect.

[8] Galluzzi F, Garavello W (2024). Treatment of peritonsillar abscess in children: a systematic review. J Clin Med.

[9] Visclosky T, Tomlinson S, Bohm L, Hashikawa A (2021). Tongue protrusion as the presenting symptom of parapharyngeal abscess. J Am Coll Emerg Physicians Open.

[10] Sousa Menezes A, Ribeiro DC, Guimarães JR, Lima AF, Dias L (2019). Management of pediatric peritonsillar and deep neck infections- cross- sectional retrospective analysis. World J Otorhinolaryngol Head Neck Surg.

[11] Tahir S, Hasanain R, Abuhammour W, Dsouza AP, Lone R, Kherani S (2023). Granulicatella adiacens causing a parapharyngeal abscess in a 10-month-old infant: a rare-case report and literature review of deep neck infections (DNIS) in children. Cureus.

[12] Rehman AU, Khan S, Abbas A, Pasha HA, Abbas Q, Siddiqui NU (2023). Life-threatening complication of retropharyngeal abscess in an infant: a case report. J Med Case Rep.

[13] Baglam T, Binnetoglu A, Yumusakhuylu AC, Gerin F, Demir B, Sari M (2015). Predictive value of the neutrophil-to-lymphocyte ratio in patients with deep neck space infection secondary to acute bacterial tonsillitis. Int J Pediatr Otorhinolaryngol.

[14] Ban MJ, Jung JY, Kim JW, Park KN, Lee SW, Koh YW, Park JH (2018). A clinical prediction score to determine surgical drainage of deep neck infection: A retrospective case-control study. Int J Surg.

[15] Koç RH, Abakay MA, Sayın İ (2024). Determining the prognostic value of CRP and neutrophil lymphocyte ratio in patients hospitalized for deep neck infection. Braz J Otorhinolaryngol.

[16] Treviño-Gonzalez JL, Acuña-Valdez F, Santos-Santillana KM (2024). Prognostic value of systemic immune-inflammation index and serological biomarkers for deep neck infections. Med Oral Patol Oral Cir Bucal.

[17] Bory C, Bory O, Guelfucci B, Nicollas R, Moreddu E (2023). Deep cervical abscesses in children: efficacy of the cefotaxime-rifampicin combination. Eur J Pediatr.

[18] Vural C, Gungor A, Comerci S (2003). Accuracy of computerized tomography in deep neck infections in the pediatric population. Am J Otolaryngol.

[19] Daya H, Lo S, Papsin BC (2005). Retropharyngeal and parapharyngeal infections in children: the Toronto experience. Int J Pediatr Otorhinolaryngol.

[20] Caprioli S, Tagliafico A, Fiannacca M (2023). Imaging assessment of deep neck spaces infections: an anatomical approach. Radiol Med.

[21] Tschauner S, Zellner M, Pistorius S, Gnannt R, Schraner T, Kellenberger CJ (2021). Ultra-low-dose lung multidetector computed tomography in children - approaching 0.2 millisievert. Eur J Radiol.

[22] Garca MF, Budak A, Demir N, Cankaya H, Kiroglu AF (2014). Characteristics of deep neck infection in children according to weight percentile. Clin Exp Otorhinolaryngol.

[23] Beka D, Lachanas VA, Doumas S, Xytsas S, Kanatas A, Petinaki E, Skoulakis C (2019). Microorganisms involved in deep neck infection (DNIs) in Greece: detection, identification and susceptibility to antimicrobials. BMC Infect Dis.

[24] Galioto NJ (2017). Peritonsillar abscess. Am Fam Physician.

[25] Tansey JB, Hamblin J, Mamidala M, Thompson J, Mclevy J, Wood J, Sheyn A (2020). Dexamethasone use in the treatment of pediatric deep neck space infections. Ann Otol Rhinol Laryngol.

